# Understanding preclinical and clinical immunogenicity risks in novel biotherapeutics development

**DOI:** 10.3389/fimmu.2023.1151888

**Published:** 2023-05-12

**Authors:** Zuben E. Sauna, Vibha Jawa, Sathy Balu-Iyer, Narendra Chirmule

**Affiliations:** ^1^ Hemostasis Branch, Division of Plasma Protein Therapeutics, Center for Biologics Evaluation and Research, Food and Drug Administration, Silver Spring, MD, United States; ^2^ Disposition and Bioanalysis, Bristol Myers Squibb (BMS), Princeton, NJ, United States; ^3^ Department of Pharmaceutical Sciences, University at Buffalo, The State University of New York, Buffalo, NY, United States; ^4^ Symphonytech Biologics, Philadelphia, PA, United States

**Keywords:** aggregates, cell-based therapies, innate immune-response modulating impurities, vaccines, TNFA polymorphisms, JAK kinase inhibitors

## Abstract

Immunogenicity continues to pose a challenge in the development of biotherapeutics like conventional therapeutic-proteins and monoclonal antibodies as well as emerging modalities such as gene-therapy components, gene editing, and CAR T cells. The approval of any therapeutic is based on a benefit-risk evaluation. Most biotherapeutics address serious medical conditions where the standard of care has a poor outcome. Consequently, even if immunogenicity limits the utility of the therapeutic in a sub-set of patients, the benefit-risk assessment skews in favor of approval. Some cases resulted in the discontinuation of biotherapeutics due to immunogenicity during drug development processes, This special issue presents a platform for review articles offering a critical assessment of accumulated knowledge as well as novel findings related to nonclinical risks that extend our understanding of the immunogenicity of biotherapeutics. Some of the studies in this collection leveraged assays and methodologies refined over decades to support more clinically relevant biological samples. Others have applied rapidly advancing methodologies in pathway-specific analyses to immunogenicity. Similarly, the reviews address urgent issues such as the rapidly emerging cell and gene therapies which hold immense promise but could have limited reach as a significant number of the patient population could potentially not benefit due to immunogenicity. In addition to summarizing the work presented in this special issue we have endeavored to identify areas where additional studies are required to understand the risks of immunogenicity and develop appropriate mitigation strategies.

## Introduction

Immunogenicity has been a central challenge in the development of biotherapeutics, which includes conventional therapeutic-proteins and monoclonal antibodies as well as emerging modalities such as components of gene therapy, gene editing, and CAR T cells. The approval of any therapeutic is based on a benefit-risk evaluation. Most biotherapeutics address serious medical conditions where the standard of care has a poor outcome. Consequently, even if immunogenicity limits the utility of the therapeutic in a sub-set of patients, the benefit-risk assessment skews in favor of approval for licensure. A few cases have resulted in the discontinuation of biotherapeutics development due to immunogenicity in the clinical trials. There continues to be an inconsistency in immunogenicity assessment strategy despite known risks of immune responses to biologics that can render them ineffective. Hence, immunogenicity continues to be a challenge in the clinic and characterization of immunogenicity and safety continues to be an issue during advance clinical development.

Understanding the mechanism of immunogenicity is critical to 1) the development of precise and accurate methods to measure antibody and T cell responses, 2) design novel molecules to overcome the immune-modulatory effects, and 3) develop immune suppression/modulatory regimes to overcome the adverse effects of unwanted immune responses. Over the past three decades a critical mass of guidance documents from different regulatory agencies, white papers from industry-regulatory agency collaborations and research reports from academia and industry have delineated the causes, consequence, and clinical effects of immune responses to biotherapeutics. This special issue presents a platform for review articles offering a critical assessment of accumulated knowledge as well novel findings related to nonclinical risks that extend our understanding of the immunogenicity of biotherapeutics. Some of the studies in this collection leveraged assays and methodologies refined over decades to more clinically relevant biological samples. Others have applied rapidly advancing methodologies in pathway-specific analyses to immunogenicity. Similarly, the reviews address urgent issues such as the rapidly emerging cell and gene therapies which hold immense promise but could have limited reach as a significant number of the potential patient population could potentially not benefit due to immunogenicity. In addition to summarizing the work presented in this special issue we have endeavored to identify areas where additional studies are required to understand the risks of immunogenicity and develop appropriate mitigation strategies.

This review summarizes the manuscripts in this special issue of the journal, which include critical quality attributes, genetic factors, immunogenicity to cell, gene therapy and gene editing modalities, immunogenicity to therapeutic proteins targeting autoimmune diseases and COVID-19 vaccines

### Critical quality attributes

The article by Swanson et al. offers a subtle but important advance on one of the most important product related factors associated with the immunogenicity of therapeutic proteins, i.e., aggregates. Foundational work on studying aggregates, over several decades, relied on artificial stress methods to induce high levels of aggregation. While this was a necessary phase in the development of appropriate assays and methodologies, artificially generated aggregates have limited biological or clinical utility. The study reported here is one of the first of its kind that evaluates spontaneously generated aggregates during the manufacturing process and during storage. An important finding was that such aggregates elicited innate immune responses for several donors in a PBMC assay with cytokine and chemokine production as a readout for immune activation. On the other hand, no significant adaptive immune responses were detected. Additionally, the extent of antibody aggregation occurring at process relevant levels are much lower than those induced through artificial stress. It is imperative that this novel approach is more widely applied to gain a better understanding of the clinical consequences of aggregation and the most appropriate methods utilized to assess such quality attributes during drug development.

While aggregates generally involve the protein/active ingredient itself, a large body of literature has established that product- and process-related impurities often function as adjuvants that activate the local or systemic innate immune response which increase the likelihood of an immune response to the active protein ingredient. The rationale for identifying/assessing innate immune response modulating impurities (IIRMIs) is clear. Unfortunately, identifying trace levels of individual IIRMI can be difficult and testing individually for all potential impurities is not feasible and cell-based assays that use human blood cells or monocyte-macrophage reporter cell lines are frequently exploited to detect minute quantities of impurities capable of eliciting innate immune activation. An important technical challenge associated with these assays is addressed in the report by Thacker et. al., i.e., excipients could blunt the cell responses, masking the presence of immunogenic IIRMI. The study painstakingly explored the impact of frequently used excipients (non-ionic detergents, sugars, amino acids, bulking agents) on the sensitivity of reporter cell lines (THP-1- and RAW-Blue cells) and fresh human blood cells. An important finding was that although excipients do not modulate the innate immune response elicited by TLR agonists *in vivo*, they do affect the sensitivity of the cell-based assays. Additionally, the investigators found that detection of model IIRMIs was negatively impacted when they tested three representative biotherapeutics: a monoclonal antibody, a growth factor, and a peptide. This report like the one previously discussed brings a new insight and suggests approaches for ensuring that assessments that are routinely carried out to evaluate immunogenicity are more meaningful.

### Genetic factors

While the articles described above focus on extant methods and approaches to extract more clinically useful information, the report by Wang et al. exploits the advances in identifying genetic variabilities associated with clinical outcomes to better understand immunogenicity. TNF inhibitors are now the standard of care in treating many autoimmune diseases such as ankylosing spondylitis, and rheumatoid arthritis. The study investigated the role of TNF-α gene polymorphisms in concert with neutrophil-to-lymphocyte ratio and platelet-to-lymphocyte ratio in predicting the efficacy and safety of TNF inhibitor therapy. Using samples from 515 subjects the study was successful in identifying biomarkers and defining a measure (TNF-α 308G/A polymorphism with neutrophil-to-lymphocyte ratio and platelet-to-lymphocyte ratio) to predict the responsiveness and safety of anti-TNF therapy in patients.

## Immunogenicity to cell, gene therapy and gene editing modalities

In addition to the original research described above, the special issue includes several topical reviews. The first of these addresses a key emerging issue in the field of immunogenicity. Emerging modalities such as gene-, CRISPR- and cell- based therapies have been demonstrated to offer treatment options for many currently intractable diseases and hold immense promise. The premise that unwanted immune responses to these novel modalities could severely compromise the safety and efficacy of these novel modalities is generally accepted. However, the presentation of the “active protein(s) component” is very different from that of conventional protein replacement and monoclonal therapies and it is not clear to what extent learnings, reagents, and methodologies applicable to protein therapies will be applicable to these modalities. For instance, it is estimated that about 20-70% of the population have pre-existing antibodies against viral vector capsids and their serotypes. In addition, development of *de novo* Anti-drug Antibodies (ADA) against vector capsids and transgene immunogenicity poses significant challenges. The gene editing CRISPR/Cas9 machinery faces similar challenges in terms of pre-existing and *de novo* immune responses. These immune responses extended to Cell based therapies that utilizes gene modification techniques, thus complicating the chemistry, manufacturing, and controls requirements. These modalities activate both innate and adaptive arms of the immune system. The vector capsids activate host innate immune system through Toll-like receptors and pathogen associated molecular patterns PAMPs. The complement system of the innate arm can be activated by immune complexes formed with pre-existing anti capsid or Cas9 antibodies. The genetic material present in these modalities can also activate Toll-like receptors. Humoral responses elicited against proteins, viral capsids and transgene can neutralize and/or accelerate clearance of these modalities. In addition to humoral response, cellular responses against gene therapy vectors and transgene proteins can be developed by presentation *via* MHC I pathway leading to generation of CD8+ cytotoxic T-cells. Further, the role of natural killer cell activation against novel modalities is emerging. The factors influencing immunogenicity are broadly classified into three categories, patient, product, and treatment related factors. These classifications cover patient variability, disease status, presence of impurities and aggregates, duration of treatment and routes of administration.

Unlike the immunogenicity of therapeutic proteins, understanding the immune responses to novel modalities is a nascent field. Khan et al. have provided a comprehensive review of the current state-of-the-art with respect to immunogenicity issues related to Chimeric antigen receptor T cell (CAR-T) therapy. CAR-T cells have provided long-term remission of a few hematological malignancies but could prove to be a very powerful weapon in the oncologist’s armamentarium. Although the six approved CAR-T therapies have yielded impressive response rates, immunogenicity could be a key challenge in the broad application of this technology. This is because the scFv domain of CAR construct, is of non-human origin in majority of the CAR-T products.

Khan et. al., have highlighted the plausible drivers of immune responses to CAR T cells and reviewed (the admittedly few studies currently published). Based on our understanding of immunogenicity from other therapeutic areas, the authors have suggested mitigation strategies to limit the immunogenic potential of CARs and improve therapeutic outcomes. Some of the key learnings from work related to novel modalities is the lack of guidance around development of novel cell and gene therapies, need for novel assay platforms as well as innovative strategies to leverage existing platforms to support preclinical and clinical development. The risks and associated critical quality attributes may impact innate and cellular responses apart from the conventional humoral response.

## Immunogenicity to therapeutic proteins targeting autoimmune diseases

In addition to original research and reviews that directly address issues related to the immune responses to therapeutic proteins, this issue also includes articles related to the treatment modalities associated with autoimmune diseases. This is because autoimmune diseases also result from unwanted immune responses to self-proteins and the progress in understanding and circumventing autoimmunity provide insights important to using small molecule drugs to modulating the immunogenicity of biopharmaceuticals. Qi et al. have provide a review of the Janus Kinase inhibitors in the treatment of the Vitiligo (a skin disorder characterized by white patches resulting from the destruction of melanocytes). Activated CXCR3+ CD8+ T cells promote melanocyte detachment and apoptosis through interferon-gamma (IFN-g secretion and chemokines secreted by keratinocytes through the Janus kinase (JAK)/signal transducer and activator of transcription (STAT)-1 signaling pathway results in further recruitment of CXCR3+ CD8+ T cells and the formation of a positive-feedback loop. JAK inhibitors target the JAK/STAT pathway and used to treat many immune-related diseases. The review discusses the success in using JAK inhibitors in targeting the interferon-γ-chemokine signaling axis in the pathogenesis and more importantly they provide a granular discussion of the molecular pathways involved in the breakdown of immune tolerance and how these may be targeted to modulate the immune responses.

## COVID-19 vaccines

The final article in this collection discusses immunogenicity in the context of vaccines where the immune response is a desirable outcome. The article is included because it provides an important insight. In combating diseases developing a medication is only part of the solution. Patient engagement “buy” in, societal narratives all have a role to play when complex decisions are made. As extraordinarily expensive treatment options like gene therapy and gene editing go mainstream it will be important to counter alternate narratives and develop systematic research strategies to understand the role of human and societal factors in the adoption of complex and controversial medications. Over the past sixty years, vaccines have prevented many infectious diseases and changed the management of public health in the world. More than thirty vaccines against various infectious diseases have been approved and ~240 vaccines are in various stages of development. National health policies in most countries provide vaccines to newborn children, adolescents, adults, and the elderly have saved millions of lives. More than 2 million lives are saved each year by vaccinations, which have resulted in the reduction of Infant Mortality from 93 deaths per 1,000 live births in 1990 to 39 in 2018.

Development of a safe, reliable, and effective vaccine was critical to curb the global COVID-19 pandemic. The end point of the COVID-19 pandemic is protection against severe disease and mortality and eventually Herd Immunity. This outcome can be achieved only after widespread availability of an effective vaccine. Many of these vaccines have been developed and approved in unprecedented timelines. Keeping track and communicating the advancements in COVID-19 vaccine development across all the platforms, monitoring the efficacy and safety relative to the circulating strains of the virus has been an astronomical task. Shi et al. have provided one of the first systematic studies of “vaccine fatigue” described as people’s inertia or inaction towards vaccine information instruction due to perceived burden and burnout. The authors identified 37 articles from PubMed, Scopus and PsycINFO, on vaccine fatigue based on pre-defined criteria. Their study found that vaccine fatigue was reported most frequently at the pre-vaccination stage. Several reasons for the vaccine fatigue included limitations of reporting of detailed vaccine sciences, effective and empathetic vaccine communications, people’s inaction towards the frequent diverse instructions from authorities. This study underscores the importance of effective communication of science to the public and is as relevant to so-called vaccine fatigue as it is to the many emerging therapies that are the subject of this collection.

## Looking to the future of immunogenicity studies

### Artificial intelligence and machine learning

It has been several decades since immunogenicity was identified as an important hurdle in the development and licensure of biotherapies. Since then, a critical mass of data has accumulated in the literature. Several disease-specific repositories also collate clinical data on immunogenicity and genomic data for individual patients. Drug Manufactures too have large unpublished data sets from different stages of the drug development. These large and complex data sets are ideal for the application of Artificial Intelligence(AI) and Machine Learning(ML) approaches to identify genetic and non-genetic risk factors associated with immunogenicity. Using training data, AI and ML can be leveraged to uncover patterns in an unbiased manner and allow the identification of biomarkers that could be used in predictive algorithms. Most importantly unlike classical statistical methods which deduce relations from data, AI and ML “learn” from the data-itself even in the absence of hypotheses to test.

### Real world evidence studies in immunogenicity

As we have alluded to above, most biotherapeutics are approved and used in the clinic even though immunogenicity issues were identified during phase 3 clinical trials. This is because the biotherapeutics address serious (often life-threatening conditions) and there are no alternatives. However, post-approval, the immunogenicity of individual biotherapeutics is not consistently monitored. There is an unmet need for real world studies on immunogenicity to better manage immunogenicity in the clinic. Real-world evidence can be generated by diverse study designs and the importance of such studies is increasingly being recognized. For instance, the 21st Century Cures Act required the FDA to expand the role of real-world evidence. Real world evidence is important because clinical trials carried out to obtain licensure do not account for the entire patient population as some geographic and/or ethnic and racial groups may have been excluded. Similarly, the genetic variability of the genes involved in the response or adverse events (e.g., immunogenicity) is not fully captured in the study population. The quality of data is a critical issue in real world evidence and this needs to be addressed prior to carrying out these studies.

### New technologies to measure immune responses; systems immunology approaches

The repertoire of technologies that can be exploited to study immunogenicity is expanding rapidly. Some of these technologies are developed in closely related fields (e.g., autoimmune diseases) and can be leveraged to obtain much more granular information about the immune response to therapeutic proteins. Technologies such as MHC Associated Peptide Proteomics and single cell RNA sequencing are more accessible in terms of both cost and the level of expertise required. The large data sets obtained from the application of these technologies are amenable to Machine Learning tools and systems immunology approaches. Such learnings will eventually be useful in modulating and managing immunogenicity during drug development (by generating less immunogenic variants) and in the clinic (by developing strategies to modulate the immune response to the therapeutic).

Taken together, the publications in this collection and emerging trends, indicate that our understanding of the immunogenicity is likely to expand exponentially soon. This will be welcome news for millions of patients who currently do not fully benefit from biotherapeutics and who would otherwise not be eligible for the promising advances from novel modalities such as gene and cell therapies.

## Data availability statement

The original contributions presented in the study are included in the article/supplementary material. Further inquiries can be directed to the corresponding author.

## Author contributions

ZS, VJ, SB and NC all contributed equally to the writing. All authors contributed to the article and approved the submitted version.

